# Behavioral Activation and Mobile Phone Addiction in Chinese University Students: The Chain-Mediating Effects of Perceived Social Support and Negative Cognitive Emotion Regulation

**DOI:** 10.3390/bs16101783

**Published:** 2026-10-01

**Authors:** Junqing Li, Youwei Yan

**Affiliations:** 1 School of Psychology, Fujian Normal University, Fuzhou 350118, China; junqing@fjut.edu.cn; 2 School of Marxism, Fujian University of Technology, Fuzhou 350118, China

**Keywords:** behavioral activation, perceived social support, negative cognitive emotion regulation, mobile phone addiction tendency

## Abstract

Behavioral activation refers to specific behavioral responses generated by individuals when exposed to reward stimuli in order to obtain reward satisfaction, and continuous rewarding experiences may lead to addictive tendencies. To understand the relationship between behavioral activation and mobile phone addiction tendency among Chinese college students, as well as the chain mediating roles of perceived social support and negative cognitive emotion regulation, a questionnaire survey was conducted among 648 students randomly sampled from a university in Fuzhou, Fujian Province, China, to investigate the relationships among behavioral activation, perceived social support, negative cognitive emotion regulation, and mobile phone addiction tendency. The results showed that: (1) behavioral activation negatively predicted mobile phone addiction tendency among Chinese college students (β = −0.11, t = −2.92, *p* < 0.001); (2) negative cognitive emotion regulation partially mediated the relationship between behavioral activation and mobile phone addiction tendency; and (3) perceived social support and negative cognitive emotion regulation played a chain mediating role between behavioral activation and mobile phone addiction tendency. These findings suggest that behavioral activation can negatively predict mobile phone addiction tendency through perceived social support and negative cognitive emotion regulation in Chinese college students.

## 1. Introduction

Chinese college students have high rates of mobile phone use and are a susceptible population for mobile phone addiction (Jin, 2024). The number of Chinese college students with mobile phone addiction showed a significant upward trend from 2013 to 2022 (Lyu et al., 2024), and China has become one of the countries with the most severe mobile phone addiction in the world (Olson et al., 2022). Mobile phone addiction generally refers to a pattern of excessive or uncontrolled smartphone use (Q. X. Liu et al., 2017), which leads to a series of emotional and behavioral problems (Sodhani et al., 2020; Y. Liu et al., 2023). In recent years, the rise of short-video applications such as TikTok has further stimulated people’s motivation to use mobile phones for entertainment (Ewing et al., 2023). However, excessive mobile phone use can affect college students’ physical and mental health and may even affect their sense of well-being (Nienstedt et al., 2023). Revealing the causes and mechanisms of mobile phone addiction tendency is conducive to addressing the problem of mobile phone addiction. Uses and Gratification Theory emphasizes that the motivation for using media such as mobile phones is to satisfy personal needs and desires (Bae, 2002). Therefore, mobile phone addiction tendency may be the result of compensatory satisfaction (Jiang et al., 2022). When individuals find that the Internet can compensate for their needs to be cared for and recognized in real life, they are prone to becoming immersed in the Internet, leading to mobile phone addiction tendency. The reinforcement sensitivity theory of personality emphasizes individual sensitivity to reward and punishment stimuli in the environment, including the behavioral activation system (BAS) and the behavioral inhibition system (BIS) (Peter et al., 2021), and provides an important theoretical framework for understanding how personality affects addictive behaviors (Corr, 2004).

### 1.1. The Relationship Between Behavioral Activation and Mobile Phone Addiction Tendency

Mobile phone addiction tendency is regulated by two major mechanisms: the behavioral activation system (BAS) and the behavioral inhibition system (BIS) (Cha & Kim, 2023). Behavioral activation refers to specific behavioral responses generated by individuals when exposed to reward stimuli in order to obtain reward satisfaction, often accompanied by positive emotional experiences. As a tool for competitive games and social platforms, mobile phones are an important source of reward stimuli. College students with high levels of behavioral activation are more likely to find pleasure and successful experiences in online games, and high behavioral activation has a significant positive predictive effect on online game addiction among college students (Xie et al., 2016). However, some studies have pointed out that college students, because they lack interest in ordinary novel things around them and have low behavioral activation levels, obtain less reward satisfaction in real life, which increases mobile phone use and leads to mobile phone addiction tendency (L. Li, 2016). Behavioral activation is negatively correlated with mobile phone addiction tendency (Mohammadi Tahroodi & Rajabpour, 2025). Moreover, mobile phone addiction tendency may, through stress, in turn drive behavioral activation, forming a vicious cycle (M. Zhang et al., 2025). Current research is mainly cross-sectional and mostly focuses on behavioral activation as an antecedent variable predicting mobile phone addiction tendency, with preventing the occurrence of mobile phone addiction tendency as the research goal. This study argues that college students with high behavioral activation levels are often able to satisfy their individualized needs through daily social interactions, invest less time and energy in mobile phone use, and have lower levels of mobile phone dependence. Therefore, behavioral activation may negatively predict mobile phone addiction tendency among Chinese college students.

### 1.2. The Mediating Role of Negative Cognitive Emotion Regulation

The internal mechanisms by which behavioral activation affects mobile phone addiction tendency still lack sufficient evidence (Gao et al., 2022). College students with low behavioral activation levels engage in fewer social activities, which can easily lead to social anxiety and depression, and low behavioral activation negatively predicts mobile phone addiction tendency (Sun et al., 2026). Compensatory Internet use theory holds that mobile phone addiction tendency is a dysfunctional emotion regulation pattern (Shahidin et al., 2022). Individuals’ negative emotion regulation patterns lead them to seek psychological compensation through Internet use, resulting in mobile phone addiction tendency (Chu et al., 2020). The Interaction of Person-Affect-The I-PACE model (Brand et al., 2016) provides a relatively comprehensive theoretical framework for understanding addictive behaviors. It highlights that addictive behaviors are shaped by the dynamic interplay of multiple factors, including affect, cognition, and executive function. According to this model, individuals who rely on negative cognitive emotion regulation strategies may become trapped in negative emotions and find it difficult to disengage from them, which can, in turn, contribute to addictive tendencies (Wang, 2021).

The cognitive theory of emotion holds that emotions arise from individuals’ cognitive appraisal of stimulus situations, emphasizing the dominant role of cognitive factors in emotion formation (J. Li, 2015). Therefore, this study introduces the concept of cognitive emotion regulation, which emphasizes the cognitive efforts individuals make to achieve emotion regulation goals when facing life events that exceed their resources (Garnefski & Kraaij, 2007). It is divided into positive cognitive emotion regulation and negative cognitive emotion regulation (Bailly et al., 2023). College students with low behavioral activation levels, when encountering negative life events, adopt negative cognitive emotion regulation strategies such as self-blame and rumination, leading to a tendency toward mobile phone addiction. Existing studies have shown that behavioral activation significantly predicts negative cognitive emotion regulation (Y. Z. Li et al., 2013). Negative cognitive emotion regulation is significantly correlated with mobile phone addiction tendency (E. L. Li, 2020). A study of community-dwelling older adults showed that negative cognitive emotion regulation partially mediated the relationship between behavioral activation and depression and anxiety (Sun, 2019). Depression and anxiety are emotional triggers of mobile phone addiction tendency. Therefore, negative cognitive emotion regulation may mediate the effect of behavioral activation on mobile phone addiction tendency among college students.

### 1.3. The Chain Mediating Role of Perceived Social Support and Negative Cognitive Emotion Regulation

Perceived social support refers to the emotional experience and degree of satisfaction of being respected, understood, and supported by others in society, that is, an individual’s subjective perception of their own level of social support (Ye et al., 2014). Individuals with high behavioral activation levels are more likely to perceive support from others or society (Deng et al., 2025). Chen et al. (2017) found that the higher an individual’s level of perceived social support, the stronger their stress resistance, and the less depression they exhibit when encountering negative events. S. Zhang et al. (2017) showed that perceived social support is positively correlated with adaptive strategies in cognitive emotion regulation and negatively correlated with maladaptive strategies. Han and Feng (2020), in a study of college students, found that perceived social support positively predicts positive cognitive emotion regulation. In summary, perceived social support may play a mediating role in the effect of behavioral activation on negative cognitive emotion regulation. Specifically, college students with low behavioral activation, due to insufficient sensitivity to external rewards, reduce social interactions, leading to lower levels of perceived social support; when encountering negative events, they are more likely to adopt negative cognitive emotion regulation strategies. Therefore, perceived social support and negative cognitive emotion regulation may play a chain mediating role between behavioral activation and mobile phone addiction tendency among Chinese college students.

**Figure 1:**
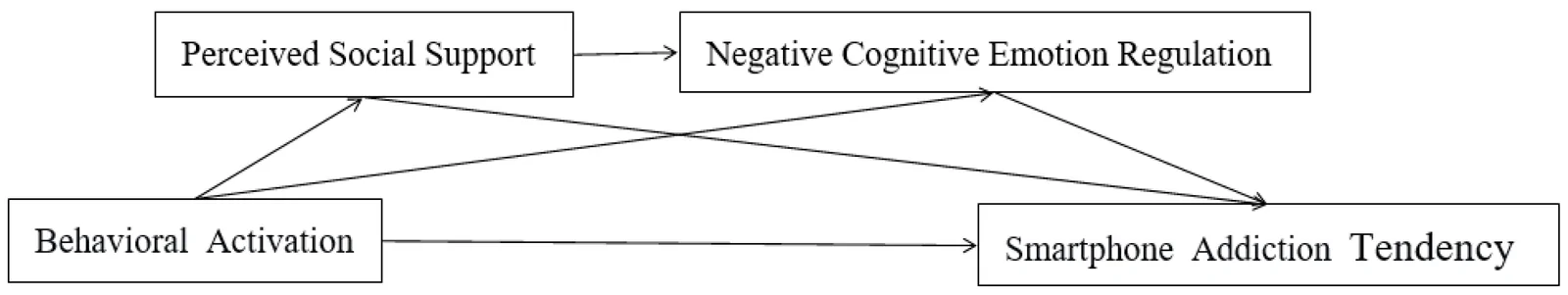
Hypothesized Model.

Based on the above review, the relationship between behavioral activation and mobile phone addiction tendency among Chinese college students may be subject to the chain mediating effects of perceived social support and negative cognitive emotion regulation. To explore these mechanisms, we propose the following hypotheses and Figure 1:

**Hypothesis** **1 (H1).** *Behavioral activation negatively predicts mobile phone addiction tendency among Chinese college students.*

**Hypothesis** **2 (H2).** *Negative cognitive emotion regulation mediates the relationship between behavioral activation and mobile phone addiction tendency among Chinese college students.*

**Hypothesis** **3 (H3).** *Perceived social support and negative cognitive emotion regulation play a chain-mediating role between behavioral activation and mobile phone addiction tendency among college students.*

## 2. Methods

### 2.1. Participants

Before data collection, informed consent was actively obtained from the school, students, and teachers. The online test was conducted according to a standardized protocol, and all relevant personnel completed standardized training. All measurements were completed in a single session, lasting between 3 and 5 min. This study adopted a cross-sectional design, which can reveal associations between variables but cannot determine causality or assess changes in variables over time. Nevertheless, this study is still of research significance for exploring the relationship between behavioral activation and mobile phone addiction tendency.

This study used random sampling. Research advertisements were distributed at a university in Fuzhou, Fujian Province, China, and only undergraduate students were eligible to participate. College students who participated in the online research tasks could receive a certain monetary reward (randomly 1–10 yuan). The study was conducted through software provided by Wenjuanxing Company. A total of 648 people filled out the questionnaire. Nine questionnaires were incomplete, and 11 questionnaires selected the same option for all items. Therefore, 628 valid questionnaires were collected, with an effective rate of 96.9%. The participants were aged 18–24 years (M = 20.67; SD = 1.22); there were 305 males and 323 females; 194 were freshmen, 153 sophomores, 148 juniors, and 133 seniors.

### 2.2. Measures

#### 2.2.1. Behavioral Inhibition/Activation System Scale

The Behavioral Inhibition/Activation System Scale was developed by Carver and White in 1994 based on Gray’s theory (Z. Q. Li et al., 2023). It is divided into the behavioral inhibition system and the behavioral activation system. This study used the revised version by Y. Z. Li et al. (2008). It contains 18 items and uses a 4-point Likert scale, ranging from 1 (strongly disagree) to 4 (strongly agree). In this study, the Cronbach’s α reliability coefficient of the scale was 0.944, with the behavioral activation subscale having a Cronbach’s α of 0.925 and the behavioral inhibition subscale having a Cronbach’s α of 0.861. **This study used only the Behavioral Activation subscale.**

#### 2.2.2. Perceived Social Support Scale

The Perceived Social Support Scale (PSSS) was developed by Zimet et al. in 1998 (S. Zhang et al., 2024). This study used the revised version by Chinese scholar Jiang Qianjin (Feng et al., 2018). The scale consists of 12 items and measures only perceived social support, including perceived support from family, friends, and others, with four items for each dimension. It uses a 7-point Likert scale, ranging from 1 (strongly disagree) to 7 (strongly agree). Higher scores indicate more perceived support. In this study, Cronbach’s α reliability coefficient of the scale was 0.956.

#### 2.2.3. Cognitive Emotion Regulation Questionnaire

The Cognitive Emotion Regulation Questionnaire (CERQ) examines individual differences in cognitive emotion regulation strategies from a cognitive perspective (X. X. Li & Li, 2019). This study used the Chinese version (CERQ-C) adapted by Zhu et al. (2007) from the Second Xiangya Hospital of Central South University, which is suitable for Chinese populations. The scale contains two strategies and nine corresponding dimensions. The positive strategies include acceptance, positive refocusing, refocus on planning, positive reappraisal, and putting into perspective. The negative strategies include self-blame, rumination, catastrophizing, and other-blame. The scale has 36 items and nine subscales, with four items per subscale. The higher the score on a subscale, the more likely the participant is to use that specific cognitive strategy when facing negative events. In this study, the Cronbach’s α coefficient of the questionnaire was 0.959, and the Cronbach’s α coefficient of the negative cognitive emotion regulation questionnaire was 0.925. **This study used only the Negative Cognitive Emotion Regulation Strategies subscale.**

#### 2.2.4. Mobile Phone Addiction Tendency Scale for College Students

The Mobile Phone Addiction Tendency Scale for College Students developed by Xiong et al. (2012) was used as the screening tool for participants. The scale has 16 items and is divided into four dimensions: withdrawal symptoms, salience, social comfort, and mood change. The scale uses a 5-point scoring system, and the internal consistency reliability of the total scale is 0.83. In this study, the Cronbach’s α coefficient of the scale was 0.933, and the Cronbach’s α coefficients of the four dimensions—withdrawal symptoms, salience, social comfort, and mood change—were 0.75, 0.81, 0.82, and 0.84, respectively.

### 2.3. Statistical Analysis

SPSS 20.0 and the SPSS macro program PROCESS 4.1 were used to organize and analyze the data. The bias-corrected percentile Bootstrap method recommended by Wen and Ye (2014) was used to test the significance of regression coefficients.

## 3. Results

### 3.1. Common Method Bias Test

Harman’s single-factor method was used to test for common method bias. After conducting exploratory factor analysis on all items using SPSS 20.0, a total of 13 factors with eigenvalues greater than 1 were found, explaining 71.71% of the variance. The variance explained by the first factor was 22.40%, which did not reach the 40% critical threshold, indicating that there was no serious common method bias in the data of this study.

### 3.2. Differences in Demographic Variables

This study examined the effects of demographic variables, including gender and grade, on behavioral activation, perceived social support, negative cognitive emotion regulation, and mobile phone addiction tendency among Chinese college students. The results showed that there was a grade difference in negative cognitive emotion regulation strategies (F = 6.62, *p* < 0.01). Specifically, sophomores scored significantly higher on negative cognitive emotion regulation strategies than other grades, while seniors scored the lowest, as shown in Table 1.

### 3.3. Descriptive Statistics and Correlation Analysis of Variables

Correlation analyses were conducted on demographic variables (gender and grade) and the total mean scores of the main variables. The results showed that behavioral activation was significantly and positively correlated with perceived social support, negative cognitive emotion regulation, and mobile phone addiction tendency. Grade was positively correlated with mobile phone addiction tendency, and gender was significantly and positively correlated with perceived social support. Overall, the correlation coefficients among the variables were relatively small, as shown in Table 2.

### 3.4. Mediation Effect Test

First, Model 4 of the SPSS macro PROCESS 4.1 (Hayes, 2013) was used to test the mediating role of negative cognitive emotion regulation between behavioral activation and mobile phone addiction tendency. The results showed that behavioral activation significantly and negatively predicted mobile phone addiction tendency (β = −0.11, t = −2.92, *p* < 0.001), supporting Hypothesis 1; behavioral activation significantly and negatively predicted negative cognitive emotion regulation (β = −0.31, t = −8.22, *p* < 0.001), and negative cognitive emotion regulation significantly and positively predicted mobile phone addiction tendency (β = 0.31, t = 7.95, *p* < 0.001). The bias-corrected percentile Bootstrap method revealed that the mediating effect value was 0.20, with a 95% confidence interval ranging from 0.27 to 0.45, not including 0, indicating that the mediating role of negative cognitive emotion regulation between behavioral activation and mobile phone addiction tendency was significant, and the residual direct effect of behavioral activation on mobile phone addiction tendency remained significant, supporting Hypothesis 2, as shown in Table 3.

This indicates that negative cognitive emotion regulation partially mediates the relationship between behavioral activation and smartphone addiction tendency.

### 3.5. Serial Mediation Analysis

**Figure 2:**
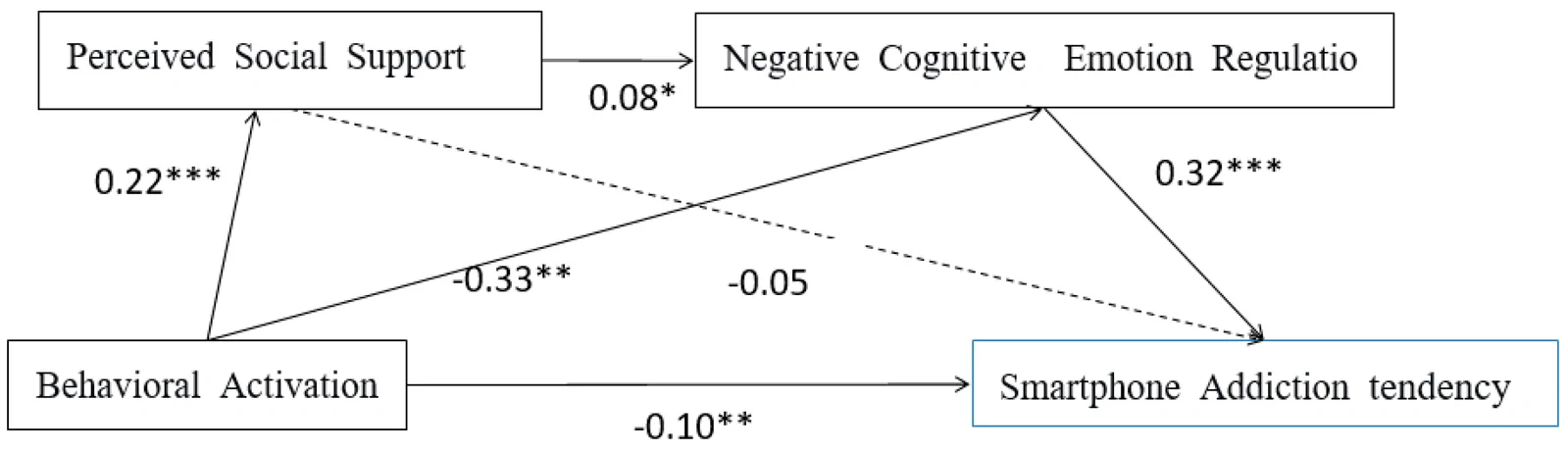
Serial Mediation Model. Note: *** *p* < 0.001,** *p* < 0.01, * *p* < 0.05.

Using PROCESS Model 6 (Hayes, 2013), we tested the serial mediating roles of perceived social support and negative cognitive emotion regulation. The bias-corrected Bootstrap method was used to determine the significance of indirect effects; if the 95% Bootstrap CI does not contain 0, the mediation effect is considered significant. As shown in Table 3, the indirect path “Behavioral Activation → Perceived Social Support → Smartphone Addiction tendency” was not significant (95% CI [−0.11, 0.02] contains 0). However, the serial mediation path “Behavioral Activation → Perceived Social Support → Negative Cognitive Emotion Regulation → Smartphone Addiction tendency” was significant (95% CI [−0.27, −0.03] does not contain 0), supporting Hypothesis 3. The final model is presented in Figure 2 and Table 4.

## 4. Discussion

### 4.1. Relationship Between Behavioral Activation and Mobile Phone Addiction Tendency Among Chinese College Students

This study explored the relationship between behavioral activation and mobile phone addiction tendency among Chinese college students and its underlying mechanisms. The findings showed that behavioral activation was negatively correlated with mobile phone addiction tendency (r = −0.21, *p* < 0.01), and behavioral activation negatively predicted mobile phone addiction tendency among Chinese college students (β = −0.11, t = −2.92, *p* < 0.001), indicating that behavioral activation may be an important predictor of mobile phone addiction tendency among college students. This result is consistent with previous research (Mohammadi Tahroodi & Rajabpour, 2025). Individuals with high levels of behavioral activation are more sensitive to reward stimuli and can keenly detect various positive stimuli in life; they are less dependent on mobile phones and are less likely to develop mobile phone addiction tendency, which is consistent with the explanation of the behavioral activation system in reinforcement sensitivity theory. **However, in this study, the correlation between behavioral activation and mobile phone addiction tendency among Chinese college students was relatively low. This may have been influenced by the sample size or the relatively concentrated distribution of participants’ responses across the response options. Further research is needed to clarify this relationship.**

### 4.2. The Mediating Role of Negative Cognitive Emotion Regulation

This study found that behavioral activation can also affect mobile phone addiction tendency through the partial mediating role of negative cognitive emotion regulation (t = 0.20), confirming Hypothesis 2 and consistent with previous research (E. L. Li, 2020). The self-emotion regulation model proposed by Bonanno (2001) suggests that individuals with difficulties in emotion regulation often satisfy their daily adaptation difficulties and self-imbalance through prolonged mobile phone use. The results of this study indicate that individuals with low behavioral activation levels tend to adopt negative cognitive emotion regulation strategies such as self-blame, catastrophizing, and rumination when encountering negative life events (Wu et al., 2021), leading to a tendency toward mobile phone addiction (He et al., 2022). Mobile phone addiction tendency is the result of failed emotion regulation, and mobile phone addiction tendency itself is an external manifestation of negative emotion regulation (Cerniglia et al., 2017). This result further validates the I-PACE model, which points out that addictive behaviors arise from the interaction among individual susceptibility (e.g., behavioral activation), affective factors (e.g., negative emotions), and cognition and affect, with the affective component playing a key role in maintaining and reinforcing addictive behaviors (Brand et al., 2016).

### 4.3. The Chain Mediating Role of Perceived Social Support and Negative Cognitive Emotion Regulation

This study confirmed that perceived social support and negative cognitive emotion regulation play a chain mediating role between behavioral activation and mobile phone addiction tendency among college students, supporting Hypothesis 3. The cognitive-affective system theory emphasizes that individual behavior is caused by different situational perceptions and cognitive processes. Individuals with low behavioral activation levels actively reduce communication with the outside world, and their perceived social support consequently decreases. When encountering negative life events, they tend to adopt negative cognitive emotion regulation strategies, leading to a tendency toward mobile phone addiction. This is consistent with the views of most studies (Feng et al., 2018; Zhao et al., 2017). Accordingly, it can also be inferred that college students with high behavioral activation levels also have high levels of perceived social support and are more likely to feel support and help from others. When encountering negative life events, the possibility of adopting positive cognitive emotion regulation strategies increases, while the need to seek social support through mobile phones decreases (H. Y. Liu et al., 2020). This study also found that the mediating role of perceived social support between behavioral activation and mobile phone addiction tendency was not significant. This may be because negative cognitive emotion regulation is a more significant mediating variable, and the mediating effect of perceived social support may be masked or diluted. Future research should further investigate the mutual influence between perceived social support and mobile phone use and explore whether mobile phone use is an important way to obtain perceived social support.

### 4.4. Limitations of This Study

First, this study adopted a cross-sectional design. Although the pathways among behavioral activation, perceived social support, negative cognitive emotion regulation, and mobile phone addiction tendency were constructed based on theory, and the stable trait of behavioral activation was explained, the causal relationships among the variables still need further verification. Future research could adopt a longitudinal tracking design to dynamically examine the long-term effects of behavioral activation on mobile phone addiction tendency among college students, thereby more accurately revealing the causal relationships. Second, the data used in this study were all derived from self-report questionnaires, with a single data source. Future research could combine other ratings, interviews, or behavioral indicators to improve the objectivity and accuracy of measurement. Third, this study took ordinary college students from two universities in Fujian as participants, which lacks representativeness and does not consider the influence of factors such as regional culture, resulting in limited applicability and stability. **Fourth, Although several correlations were observed among the main variables in this study, the correlation coefficients were generally small. This may have been due to the relatively large sample size. Future research could therefore attempt to use experimental designs to further examine these relationships**. Fifth, the data in this study were collected during the COVID-19 pandemic. Chinese college students were affected by home isolation during the pandemic, which may have further exacerbated mobile phone addiction tendency. This study did not conduct research and analysis on the relevant background.

In summary, this study, to a certain extent, revealed the internal mechanism by which behavioral activation affects mobile phone addiction tendency and further enriched the understanding of the formation mechanism of mobile phone addiction tendency. The results also suggest that in the future, platforms for communication with the outside world can be built in ways that college students enjoy, to improve their level of perceived social support. In addition, college students can be educated and guided to form reasonable cognitive emotion regulation strategies to reduce the degree of mobile phone addiction tendency.

## 5. Conclusions

The present study indicates that Chinese college students exhibit a relatively high level of mobile phone addiction tendency and that behavioral activation negatively predicts this tendency. Perceived social support and negative cognitive emotion regulation exerted a sequential mediating effect on the association between behavioral activation and mobile phone addiction tendency, thereby providing preliminary evidence for explaining the formation of mobile phone addiction tendency. These findings suggest that maintaining an appropriate level of behavioral activation may help prevent mobile phone addiction; future interventions could therefore alleviate Chinese college students’ mobile phone addiction tendency by enhancing their behavioral activation. In school education, efforts should be made to enrich campus cultural life in accordance with students’ interests and to foster harmonious interpersonal relationships to the greatest extent possible, thereby enhancing students’ perceived social support and strengthening their confidence that others and society will provide proactive help when they encounter negative events. In family education, emphasis should be placed on improving college students’ emotion regulation capacity and guiding them to adopt positive emotion regulation strategies when experiencing negative emotions, so that they can better adapt to real-world social life and indirectly reduce their mobile phone addiction tendency. Future research could recruit college students with mobile phone addiction tendency as participants to examine the causal relationship between their typical mobile phone use characteristics and behavioral activation. However, given the cross-sectional nature of the present data, definitive causal inferences cannot be drawn. To rigorously assess the efficacy of the interventions recommended herein, future research should adopt experimental or longitudinal designs.

## Figures and Tables

**Table 1:** Demographic Differences in Main Variables.

Variable	Category	Behavioral Activation (M ± SD)	Perceived Social Support (M ± SD)	Negative Cognitive Emotion Regulation Strategies (M ± SD)	Mobile Phone Addiction Tendency (M ± SD)
Gender	Male (n = 305)	8.99 ± 2.28	7.32 ± 2.09	9.11 ± 2.37	9.02 ± 2.67
	Female (n = 323)	8.71 ± 2.03	7.68 ± 2.15	9.35 ± 2.04	8.90 ± 2.42
	t	1.64	−2.14	−1.35	0.49
Grade	Freshman	9.06 ± 2.10	7.42 ± 2.10	8.95 ± 2.19	8.68 ± 2.52
	Sophomore	8.76 ± 2.20	7.66 ± 2.21	9.83 ± 2.08	8.96 ± 2.55
	Junior	8.57 ± 2.48	7.50 ± 2.26	9.37 ± 2.44	9.00 ± 2.73
	Senior	8.94 ± 1.80	7.50 ± 2.13	8.83 ± 1.97	8.95 ± 2.73
	F	1.65	0.39	6.62 **	1.53
				2 > 1 *, 2 > 4 *, 3 > 4 *, 2 > 3 *	

Note: ** *p* < 0.01, * *p* < 0.05. 1 = Freshman, 2 = Sophomore, 4 = Senior.

**Table 2:** Correlations Among Main Variables (n = 628).

Variables	$\bar{x}$ ± *s*	1	2	3	4	5	6
1. Perceived Social Support	7.50 ± 2.13	1					
2. Negative Cognitive Emotion Regulation	9.24 ± 2.21	0.01					
3. Behavioral Activation	8.85 ± 2.16	0.22 **	−0.31 **				
4. Smartphone Addiction tendency	8.95 ± 2.54	−0.07 **	0.35 **	−0.21 **			
5. Gender	1.51 ± 0.5	0.09 *	0.05	−0.07	−0.02		
6. Grade	2.35 ± 1.13	0.01	−0.02	−0.04	0.08 *	−0.22 **	1

Note: ** *p* < 0.01, * *p* < 0.05.

**Table 3:** Mediation Model Testing.

Outcome Variable	Predictor	*β*	*SE*	*t*	*p*	95% CI
Smartphone Addiction tendency	Behavioral Activation	−0.17	0.06	−2.91	<0.01	[−0.05,−0.11]
Negative Cognitive Emotion Regulation	Behavioral Activation	−0.31	0.05	−8.22	<0.01	[−0.50,−0.30]
Smartphone Addiction tendency	Behavioral Activation	−0.11	0.06	−2.92	<0.05	[−0.28,−0.05]
	Negative Cognitive Emotion Regulation	0.31	0.05	7.95	<0.01	[0.27,0.45]

**Table 4:** Serial Mediation Model Testing.

Outcome Variable	Predictor	*β*	*SE*	*t*	*p*	95% CI
Perceived Social Support	Behavioral Activation	0.22	0.07	5.60	<0.01	[0.24,0.51]
Negative Cognitive Emotion Regulation	Perceived Social Support	0.08	0.03	2.17	=0.03	[0.01,0.12]
	Behavioral Activation	−0.33	0.05	−8.52	<0.01	[−0.52,−0.33]
Smartphone Addiction tendency	Negative Cognitive Emotion Regulation	0.32	0.45	8.04	<0.01	[0.28,0.45]
	Perceived Social Support	−0.05	0.33	−1.34	=0.18	[−0.11,0.02]
	Behavioral Activation	−0.10	0.06	−2.52	=0.01	[−0.27,−0.03]

## Data Availability

The original contributions presented in this study are included in the article. Further inquiries can be directed to the corresponding author.
